# Supplementary material for: Step-like dependence of memory function on pulse width in spintronics reservoir computing
Source: Sci Rep. 2020 Nov 11;10:19536. doi: 10.1038/s41598-020-76142-x (PMC7659325; doi:10.1038/s41598-020-76142-x)
Supplement: Supplementary file 1 — Supplementary information. [file 41598_2020_76142_MOESM1_ESM.pdf]

Step-like dependence of memory function on pulse width in spintronics reservoir computing

Terufumi Yamaguchi[1], Nozomi Akashi[2], Kohei Nakajima[2], Hitoshi Kubota[1], Sumito Tsunegi[1], and Tomohiro Taniguchi[1]

[1]National Institute of Advanced Industrial Science and Technology (AIST), Spintronics Research Center, Tsukuba, Ibaraki 305-8568, Japan
[2]Graduate School of Information Science and Technology, The University of Tokyo, Bunkyo-ku, 113-8656 Tokyo, Japan

ABSTRACT

In this Supplementary information, we provide the data summarizing the dependences of the short-term memory (STM) and parity-check (PC) capacities on the training data N, and show these capacities for several values of the input-current strength ν.

1 Number of training data and values of capacities

The number of the training data N should be large enough to train the reservoir. Figure S1 shows the dependences of the STM and PC capacities on the number of the training data N, where the parameter ν determining the strength of the input data is (a) 0.05, (b) 0.10, and (c) 0.20, whereas the pulse width is 2.5 μs. Note that the number of the training data N should be larger than the node number $N_{\rm node} = 250$ to determine the Moore-Penrose inverse matrix uniquely by the singular value decomposition. The results indicate that $N = 1000$ used in the main text is suffucient to obtain saturated values of the capacities.

2 Memory capacity and input-current strength

In the main text, we show the calculated values of the STM and PC capacities for the input-current strengths of $\nu = 0.20$ and 0.05. Here, we show these capacities for a wide range of ν.

Figure S2 summarizes the dependences of the STM and PC capacities for a wide range of the parameter ν of (a) 0.005, (b) 0.01, (c) 0.05, (d) 0.10, (e) 0.15, and (f) 0.20, where Figs. S2(c) and S2(f) are identical to Fig. 3(c) and 5(b) in the main text but are shown here again for comparison. As discussed in the main text, the pulse width at which the jump from $C = 2$ to $C = 1.5$ appears is shifted to a long pulse-width range as ν increases. In addition, the pulse-width range corresponding to $C = 1.5$ nearly disappears for a sufficiently small ν ($\nu = 0.005$ in the present case) because the relaxation times from bi $= 0$ to bi $= 1$ and vice versa become nearly identical for small ν. The results support the main argument made in the main text that the step-like behaviour appears due to the current-dependent vortex relaxation. We also note that the capacities in short pulse-width ranges show rapid growth with increasing the pulse width. We consider that it arises from the competition between two

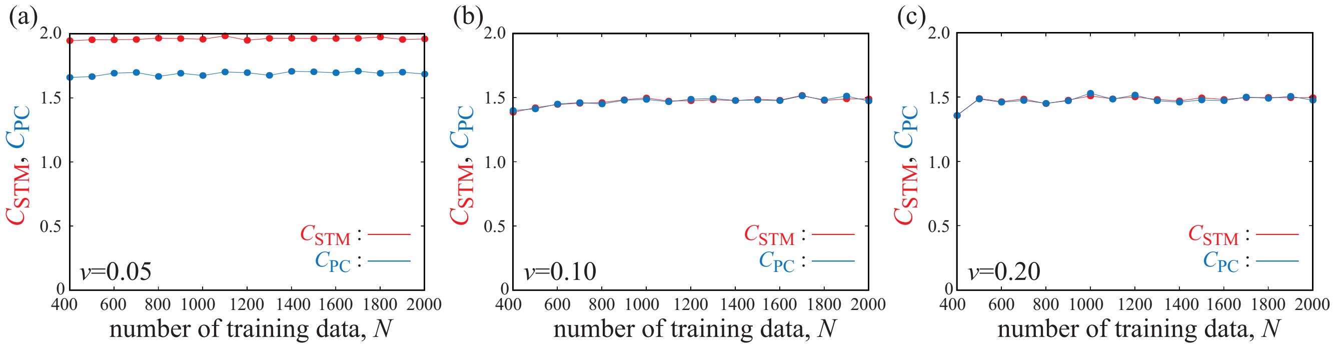

Figure S 1. Dependences of the STM (red) and PC (blue) capacities on the number of the training data N where the dimensionless parameter ν determining the difference of the magnitudes of binary inputs is (a) 0.05, (b) 0.10, and (c) 0.20, respectively. The pulse width is 2.5 μs.

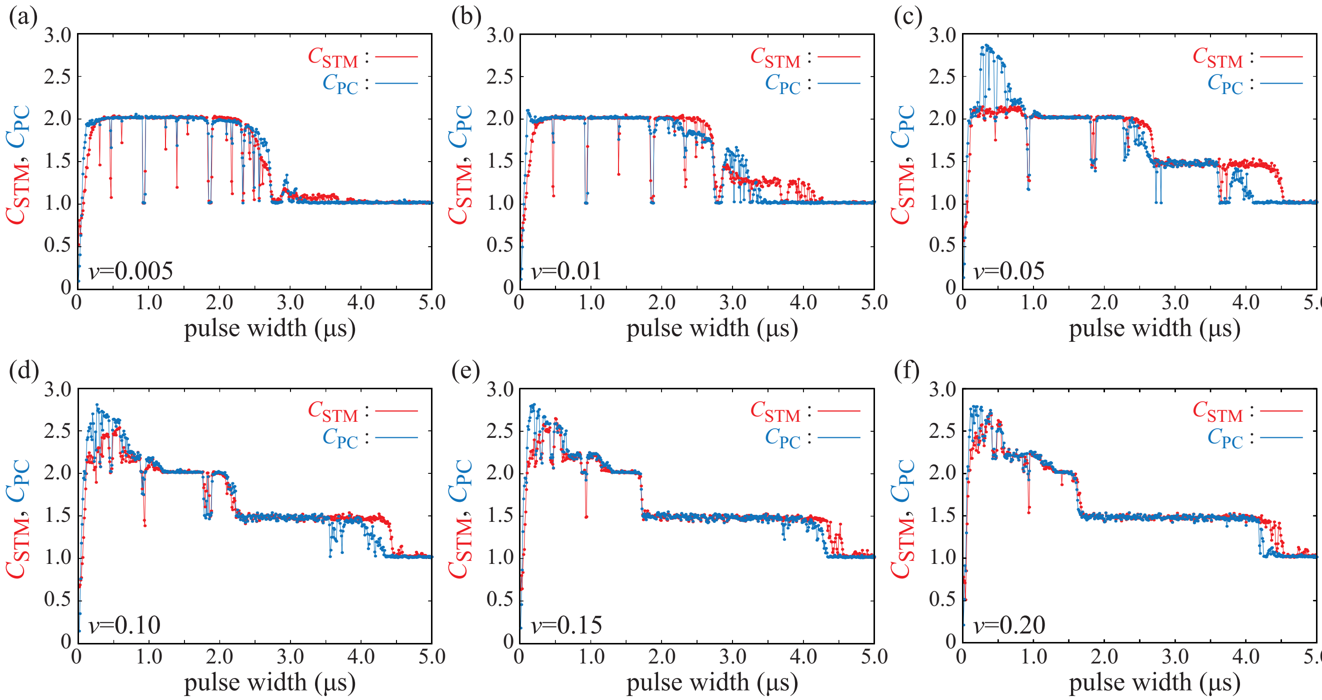

Figure S 2. Dependences of the STM (red) and PC (blue) capacities on the pulse width, where the dimensionless parameter ν determining the difference of the magnitudes of binary inputs is (a) 0.005, (b) 0.01, (c) 0.05, (d) 0.10, (e) 0.15, and (f) 0.20, respectively.

factors. First, the capacity increases with the increases of a pulse width when the pulse width is shorter than the relaxation time of the vortex core, which is about 0.36 μs for $\nu = 0.20$, as mentioned in the main text. This is because the change of the vortex core with respect to the injection of the input pulse becomes large by increasing the pulse width; if the input pulses are injected within a short time period, we could not observe a visible change of the core position during one pulse. A visible change of the core position contributes to identify the input data. Therefore, the memory capacity increases as the pulse width increases. Simultaneously, however, increasing the pulse width makes the core position saturated, which contributes to forgetting the memory of the past input data. Because of the competition between these two factors, the capacities in the short pulse-width range do not show step-like behaviour.

We note that the upper value of the parameter ν is set to be 0.20 because a further increase of the parameter makes the variable s larger than 1. Since s is the normalised distance of the vortex core measured from the disc centre, s should satisfy $s \leq 1$. The fact that s becomes larger than 1 means that the vortex core is excluded from the ferromagnet due to a large spin-transfer torque, where the phenomenon is known as a vortex-core expulsion[1,2]. It means that the Thiele equation is no longer applicable to describe the magnetisation state, and the system cannot be applied to reservoir computing because the ferromagnet becomes uniformly magnetised state without emitting time-dependent signal. We also notice that the step-like behaviour is more obvious for the STM capacity, whereas the behaviour of the PC capacity is not so simple compared with the STM capacity, which might come from the nonlinear transformation of the input data. Emphasis should be made here that the discussion in Fig. 4 in the main text focuses on the identification of the input signal without any transformation, which corresponds to that evaluated in the STM capacity. Therefore, we consider that the step-like behaviour is more obvious in the STM capacity than the PC capacity.

References

1. Jenkins, A. S. *et al.* Spin-torque resonant explusion of the vortex core for an efficient radiofrequency detection scheme. *Nat. Nanotechnol.* **11**, 360 (2016).

2. Tsunegi, S. *et al.* Acheivement of high diode sensitivity via spin torque-induced resonant expulsion in vortex magnetic tunnel junction. *Appl. Phys. Express* **11**, 053001 (2018).
